# Vorwort der Herausgeber

**DOI:** 10.1007/s11943-020-00274-9

**Published:** 2020-08-04

**Authors:** Timo Schmid, Markus Zwick

**Affiliations:** 1 Institut für Statistik und Ökonometrie, Freie Universität, Berlin, Deutschland; 2 Institut für Forschung und Entwicklung in der Bundesstatistik, Statistisches Bundesamt, Wiesbaden, Deutschland

Liebe Leserinnen und Leser des AStA Wirtschafts- und Sozialstatistisches Archiv,

leider beginnen die Artikel dieser Ausgabe mit einer traurigen Nachricht. Am 23. Dezember 2019 ist überraschend Prof. Dr. Gerd Ronning, langjähriges und sehr aktives Mitglied der Deutschen Statistischen Gesellschaft, im Alter von 80 Jahren verstorben. In Deutschland war er einer der ersten, der sich mit der Schnittstelle zwischen Statistik, Mikroökonomie und der Ökonometrie beschäftigte. Neben den Arbeiten im Bereich der Mikroökonometrie befasste er sich mit Fragen der empirischen Kapitalmarktforschung und Wirtschaftsforschung. Seit 1993 und bis zu seiner Pensionierung 2004 hatte Gerd Ronning den Lehrstuhl für Statistik, Ökonometrie und Empirische Wirtschaftsforschung an der Universität Tübingen inne und leitete zudem als Direktor das Institut für Angewandte Wirtschaftsforschung. Von 1987 bis 1999 war er (stellvertretender) Vorsitzender des Ausschusses für Empirische Wirtschaftsforschung und Angewandte Ökonometrie in der Deutschen Statistischen Gesellschaft. Ein Nachruf auf Gerd Ronning wurde von Robert Jung, Martin Kukuk und Roman Liesenfeld verfasst (Jung et al. 2020).

Mittlerweile eine wichtige Konstante im AStA Wirtschafts- und Sozialstatistisches Archiv ist die Publikation der auf der Statistischen Woche gehaltenen Heinz-Grohmann-Vorlesung. Im September 2019 stellte Bernd Fitzenberger die gemeinsame Arbeit mit Arnim Seidlitz, zum Thema der Lohnungleichheit von Vollzeitbeschäftigten in Deutschland vor, die wir in diesem Heft nun veröffentlichen (Fitzenberger und Seidlitz 2020). Die Autoren geben einen Rückblick und Überblick über die Entwicklung der Lohnungleichheit beginnend mit den achtziger Jahren des letzten Jahrhunderts. Der Schwerpunkt liegt dabei auf der Entwicklung der Lohnungleichheit für Vollzeitbeschäftigte in Westdeutschland, in ausgewählten Bereichen erweitert der Beitrag die Ergebnisse für Frauen und für Ost- und Gesamtdeutschland. Der Artikel berücksichtigt hierbei zum einen die umfangreiche einschlägige Literatur zum Thema, die Autoren wertet zum anderen aber auch die Stichprobe der Integrierten Arbeitsmarktbiografien (SIAB-Daten) des Instituts für Arbeitsmarkt- und Berufsforschung zum Thema aus. Die hierbei herausgearbeiteten zentralen Befunde sind: In Westdeutschland kam es zwischen 1980 und 2010 zu einem deutlichen Anstieg der Lohnungleichheit von Vollzeitbeschäftigten, der sich zunächst auf den oberen Bereich der Lohnverteilung beschränkte und ab Mitte der 1990er Jahre sowohl im oberen als auch im unteren Bereich der Lohnverteilung fortsetzte. Im Zeitraum 1995 bis 2010 ging die Entwicklung mit starken Reallohnverlusten im unteren Bereich der Lohnverteilung einher. Nach 2010 stieg die Lohnungleichheit für Vollzeitbeschäftigte nicht weiter an, aber es kam zu keinem deutlichen Rückgang der Lohnungleichheit. Die Reallöhne stiegen über die gesamte Lohnverteilung seit 2010 deutlich an. Die aufgezeigte Entwicklung der Lohnungleichheit von Vollzeitbeschäftigten in Westdeutschland wird dann im Folgenden hinsichtlich der teilweise sehr kontrovers diskutieren Erklärungsfaktoren erörtert.

Ein weiteres sozial brisantes Thema ist der angespannte Wohnungsmarkt. Durch die Einführung der so genannten Mietpreisbremse sollen massive Preissprünge bei der Neuvermietung von Wohnungen verhindert werden. Die Richtschnur zur Beurteilung der Verhältnismäßigkeit von Mietpreissteigerungen soll dabei die ortsübliche Vergleichsmiete sein. Ortsübliche Vergleichsmieten wiederum finden sich in örtlichen, teils qualifizierten, Mietspiegeln, die aber ihrerseits teils unter Kritik stehen. Während die aktuelle politische Debatte um qualifizierte Mietspiegel eher von der statistischen Datenauswertung zur Erstellung der qualifizierten Mietspiegel geprägt ist, legen Kauermann et al. (2020) in ihrem Artikel den Fokus auf die Datenerhebung und Stichprobenziehung bei qualifizierten Mietspiegeln. Sie analysieren die Mietspiegelpraxis der 30 größten deutschen Städte und diskutieren diese aus Sicht der Statistik. Drei Aspekte werden dabei besonders verfolgt: 1. Zur Beantwortung der Frage, wer befragt wird, erfolgt eine Gegenüberstellung von Mieter- und Vermieterbefragungen. 2. Die Vor- und Nachteile von verschiedenen Datenerhebungsformen, wie schriftliche Fragebögen, Interviews oder Kombinationen aus beiden Befragungsarten, werden untersucht. 3. Außerdem stellen die Autoren die in der Praxis angewandten Stichprobenverfahren und -designs vor und gehen vor allem auf die Problematik ein, dass vollständige Listen aller mietspiegelrelevanten Haushalte oder Wohnungen nicht vorhanden sind.

Zinn et al. (2020) geben in dieser Ausgabe des AStA Wirtschafts- und Sozialstatistisches Archiv einen Einblick in mögliche Stichprobenprobleme des Nationalen Bildungspanels (National Educational Panel Study, NEPS), in dem Informationen zu Individuen in sechs verschiedenen Kohorten mit einem Alter von 6 bis 8 Monaten bis hin zum Erwachsenenalter erfasst werden, welche wiederum eine Analyse der Kompetenzentwicklung ermöglichen. Der Beitrag gibt einen Überblick zu der Anzahl der Befragten, die an der Panelbefragung teilgenommen haben, sowie die Anzahl der Befragten, die aus dem Panel über die Zeit ausgefallen (Attrition) sind. Dabei werden insbesondere Stichprobenumfänge, Teilnehmerzahlen und temporäre und endgültige Abbrecherquoten für jede Welle einzeln betrachtet, stichprobenspezifische Besonderheiten diskutiert und mögliche selektive Verzerrungen untersucht. Eine Diskussion von konkreten Empfehlungen, die in der statistischen Analyse berücksichtigt werden sollen, um mögliche Selektionsverzerrungen und fehlerhafte Inferenz zu vermeiden, runden den Beitrag ab.

Auch in diesem Heft setzen wir die Reihe der Interviews mit prägenden deutschen Statistikerinnen und Statistikern im AStA Wirtschafts- und Sozialstatistisches Archiv fort. Dieses Mal hat Walter Krämer (2020) Christine Müller von der Technischen Universität Dortmund interviewt. Ihre akademische Ausbildung absolvierte Frau Müller an der FU Berlin. Nach dem Studium der Mathematik und anschließender Promotion im Gebiet der statistischen Versuchsplanung habilitierte Frau Müller 1995 mit einem Thema im Bereich der robusten Statistik. Nach Zwischenstationen in Göttingen, Oldenburg und Kassel lehrt und forscht sie als Professorin für Statistik in den Ingenieurwissenschaften seit zehn Jahren an der Fakultät für Statistik an der TU Dortmund. Dass sie nicht nur akademische Exzellenz, sondern auch Führungsgeschick auszeichnet, demonstrierte sie als langjährige Vorsitzende der Deutschen Arbeitsgemeinschaft Statistik (DAGStat). In Krämer (2020) gibt Frau Müller unter anderem Einblicke in ihren Werdegang und diskutiert mit Walter Krämer die Rolle von statistischen Gesellschaften in Deutschland.

Und zuletzt dürfen wir Sie, liebe Leserinnen und Leser, ermuntern, das AStA Wirtschafts- und Sozialstatistisches Archiv mit Ihren Beiträgen zu unterstützen. Vielleicht nutzen Sie dabei die Gelegenheit eine Arbeit im Sonderheft zum Thema „Covid-19“ einzureichen. Die Corona-Pandemie und ihre Folgen verändern derzeit die Situation vieler Menschen in Deutschland. Umso wichtiger sind valide statistische Analysen in diesem Bereich. Die Beiträge zu diesem vorgesehenen Sonderheft sollen deutlich machen, dass der vernünftige Umgang mit Zahlen und daraus abgeleiteten Statistiken wichtig für ein valideres Verständnis der Pandemie und deren Folgen ist. Zusätzlich können die Beiträge sich aber auch mit fehlerhaften Analysen oder Ableitungen durch fehlende Statistical Literacy und den daraus abgeleiteten Folgerungen beschäftigen. Wir als Herausgeber des AStA Wirtschafts- und Sozialstatistisches Archiv geben zusammen mit Ralf Münnich von der Universität Trier dieses Sonderheft heraus und freuen uns über Einreichungen bis zum 15. September 2020.

Nun wünschen wir Ihnen, liebe Leserinnen und Leser, viel Spaß bei der Lektüre der zweiten diesjährigen Ausgabe des AStA Wirtschafts- und Sozialstatistisches Archivs.

Timo Schmid und Markus Zwick
